# Exploration of Machine Learning and Statistical Techniques in Development of a Low-Cost Screening Method Featuring the Global Diet Quality Score for Detecting Prediabetes in Rural India

**DOI:** 10.1093/jn/nxab281

**Published:** 2021-10-23

**Authors:** Nick Birk, Mika Matsuzaki, Teresa T Fung, Yanping Li, Carolina Batis, Meir J Stampfer, Megan Deitchler, Walter C Willett, Wafaie W Fawzi, Sabri Bromage, Sanjay Kinra, Shilpa N Bhupathiraju, Erin Lake

**Affiliations:** Department of Biostatistics, Harvard TH Chan School of Public Health, Boston, MA, USA; Department of Non-Communicable Disease Epidemiology, London School of Hygiene and Tropical Medicine, University of London, London, United Kingdom; Department of Nutrition, Harvard TH Chan School of Public Health, Boston, MA, USA; Department of International Health, Johns Hopkins Bloomberg School of Public Health, Baltimore, MD, USA; Nutrition Department, Simmons University, Boston, MA, USA; Department of Nutrition, Harvard TH Chan School of Public Health, Boston, MA, USA; CONACYT—Health and Nutrition Research Center, National Institute of Public Health, Cuernavaca, Mexico; Department of Nutrition, Harvard TH Chan School of Public Health, Boston, MA, USA; Department of Epidemiology, Harvard TH Chan School of Public Health, Boston, MA, USA; Channing Division of Network Medicine, Department of Medicine, Brigham and Women's Hospital, Harvard Medical School, Boston, MA, USA; Intake—Center for Dietary Assessment, FHI Solutions, Washington, DC, USA; Department of Nutrition, Harvard TH Chan School of Public Health, Boston, MA, USA; Department of Epidemiology, Harvard TH Chan School of Public Health, Boston, MA, USA; Channing Division of Network Medicine, Department of Medicine, Brigham and Women's Hospital, Harvard Medical School, Boston, MA, USA; Department of Global Health and Population, Harvard TH Chan School of Public Health, Boston, MA, USA; Department of Nutrition, Harvard TH Chan School of Public Health, Boston, MA, USA; Department of Non-Communicable Disease Epidemiology, London School of Hygiene and Tropical Medicine, University of London, London, United Kingdom; Department of Nutrition, Harvard TH Chan School of Public Health, Boston, MA, USA; Channing Division of Network Medicine, Department of Medicine, Brigham and Women's Hospital, Harvard Medical School, Boston, MA, USA; Department of Biostatistics, Harvard TH Chan School of Public Health, Boston, MA, USA

**Keywords:** GDQS, prediabetes, machine learning, GLMM, mixed model, cluster-correlation, LASSO, random forest, survey, diabetes

## Abstract

**Background:**

The prevalence of type 2 diabetes has increased substantially in India over the past 3 decades. Undiagnosed diabetes presents a public health challenge, especially in rural areas, where access to laboratory testing for diagnosis may not be readily available.

**Objectives:**

The present work explores the use of several machine learning and statistical methods in the development of a predictive tool to screen for prediabetes using survey data from an FFQ to compute the Global Diet Quality Score (GDQS).

**Methods:**

The outcome variable prediabetes status (yes/no) used throughout this study was determined based upon a fasting blood glucose measurement ≥100 mg/dL. The algorithms utilized included the generalized linear model (GLM), random forest, least absolute shrinkage and selection operator (LASSO), elastic net (EN), and generalized linear mixed model (GLMM) with family unit as a (cluster) random (intercept) effect to account for intrafamily correlation. Model performance was assessed on held-out test data, and comparisons made with respect to area under the receiver operating characteristic curve (AUC), sensitivity, and specificity.

**Results:**

The GLMM, GLM, LASSO, and random forest modeling techniques each performed quite well (AUCs >0.70) and included the GDQS food groups and age, among other predictors. The fully adjusted GLMM, which included a random intercept for family unit, achieved slightly superior results (AUC of 0.72) in classifying the prediabetes outcome in these cluster-correlated data.

**Conclusions:**

The models presented in the current work show promise in identifying individuals at risk of developing diabetes, although further studies are necessary to assess other potentially impactful predictors, as well as the consistency and generalizability of model performance. In addition, future studies to examine the utility of the GDQS in screening for other noncommunicable diseases are recommended.

 

 

## Introduction

Type 2 diabetes (T2D) continues to increase substantially in South Asia (1) with more than half of T2D cases being undiagnosed (2). To mitigate the increasing rates of T2D, it is imperative to identify individuals with prediabetes to prevent progression to T2D. This is especially crucial in rural areas in low- and middle-income countries (LMICs) like India where over two-thirds of the population live in resource-limited, rural areas (3). Because diagnosis for prediabetes and T2D through laboratory measures can be expensive or unavailable in rural areas, lower-cost alternatives screening for higher-risk individuals can offer a strategy in halting the rise of T2D rates in resource-limited settings.

Machine learning (ML) and statistical methods may assist in discovering patterns present in data that are predictive of diabetes risk. ML techniques can be quite effective in identifying prediabetes, although many predictive algorithms currently in existence require expensive imaging technology or laboratory measurements (4). Some low-cost scoring methods using a combination of ML techniques and questionnaire data have been shown to be effective in screening for T2D (5, 6). However, similar affordable screening tools for prediabetes are currently not available, to our knowledge.

Because diet quality is a strong risk factor for T2D development (7), examining the predictive performance of low-cost dietary assessment tools with ML techniques can potentially lead to development of new screening methods for use in rural areas in LMICs. The Global Diet Quality Score (GDQS), a global measure of diet quality, is a novel, low-cost, food-based instrument for measuring diet that is easy to interpret and has been tested in a number of LMIC settings (8). The GDQS has previously been shown to be positively associated with nutrient adequacy among a cohort of nonpregnant Indian women of reproductive age (9) and also with lower T2D risk in a cohort of US women (10), suggesting that the score has potential as a screening method for prediabetes in this population when combined with ML techniques and other relevant covariates. Background information and definition of the GDQS metric referenced throughout can be found in this supplement via the feature “Development and validation of a novel food-based Global Diet Quality Score,” and thus will not be presented here.

The objective of the current study was to use several ML techniques, and compare their performance using area under the receiver operating characteristic curve (AUC), in the development of a prediction algorithm that utilizes measures of diet quality and other simple predictors of diabetes risk such as age and tobacco use that can be easily obtained from questionnaire data. The goal was to reduce the need to screen all individuals using laboratory-based measures and instead prioritize testing for individuals identified as higher-risk via a suitable prediction equation.

Our analysis includes an exploration into several well-established ML and statistical methods (including one which accounts for correlated outcomes within-family) and provides a foray into viable analysis tools when cluster-correlation (similar to longitudinal correlation) is present in the dietary/disease data under study. A predictive tool for screening prediabetes, using the GDQS metric and other risk factors as predictors, is proposed, offering a unique contribution in prediabetes classification. Although the GDQS alone can be used to assess risk of several chronic diseases, we explore how it can be used as a predictor among other variables when identifying prediabetes specifically. We compare techniques via model performance with respect to AUC [where rule-of-thumb maintains that AUC >0.70 suggests good/adequate model performance, AUC >0.80 suggests great model performance, and AUC >0.90 suggests excellent model performance (11)], sensitivity, and specificity in the test data set.

## Methods

### Cohort information and study population

The current study examines a cross-sectional set of data from the Andhra Pradesh Children and Parents Study (APCAPS). Details of the APCAPS have been published elsewhere (12). Briefly, the study's first wave of data collection in 2003–2005 examined children who were part of the Hyderabad Nutrition Trial (1987–1990), which assessed the impact of a governmental public health program on birth weight (12). These children are referred to as the APCAPS index children (*n* = 1492). Measurements were taken for this group of index children in 2003–2005 (*n* = 1492; first wave of data collection) and again in 2009–2010 (*n* = 2581; second wave). During the subsequent third wave of measurements taken in 2010–2012 (repeated measurements for the index children), the cohort expanded to include the mothers, fathers, and siblings of the index children (*n* = 8383). Because it contained the most recent information at the time of the present analysis, only data from the third wave were used in the current work. The APCAPS received approval from the ethics committees of the National Institute of Nutrition (Hyderabad, India), the Indian Council of Medical Research, and the London School of Hygiene and Tropical Medicine (London, United Kingdom).

**FIGURE 1:**
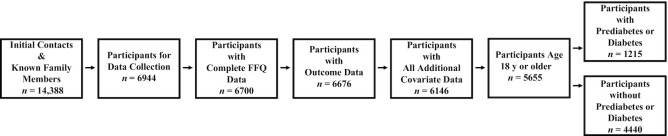
Flowchart of study sample size.

For the current analyses, we excluded family members of index children who did not participate in the third wave of data collection and index children who were lost to follow-up (*n* = 7444). We only included participants ≥18 y of age who had complete information on age, sex, blood glucose, select demographic information, and all FFQ data (*n* = 5655) (Figure 1). This final sample of 5655 individuals came from 1728 different families.

### Dietary assessment

Dietary information was collected by trained interviewers through a validated semiquantitative 98-item FFQ (12). This list of foods was a subset of a longer FFQ adapted for use in both urban and rural India (13). Participants reported how often they consumed each food in the past year, using relevant references for portion size such as bowl, ladle, or raw number at frequencies of per day, per week, per month, and per year. This questionnaire has been validated against 24-h dietary recalls and was found to have acceptable validity, with κ statistics ranging from 0.07 to 0.51 for particular food groups (13).

### GDQS

We calculated the GDQS for each individual by totaling daily intake in grams for the GDQS food groups and categorizing these into categories of “low,” “medium,” and “high” consumption. For high fat dairy, we included an additional “very high” consumption category. The points system used to compute the GDQS is included as supplementary information (**Supplemental Table 1**), but in general, a higher GDQS means higher diet quality. We disaggregated mixed dishes into individual foods which were then each classified into their corresponding GDQS food groups based upon recipes derived from participants of the Indian Migration Study (14). Owing to their geographical location, all villages in the APCAPS cohort are considered part of the rural Hyderabad region. Because no recipes from this region included foods from the deep orange tubers, juice, low fat dairy, or processed meat categories, consumption amounts for these food groups were unknown for all participants. As a result, these categories were not included in computing the final GDQS. Details of the development of the GDQS can be found in the main article of this supplement (8).

### Assessment of prediabetes

Fasting blood samples were collected in the APCAPS during follow-up and an assay was performed to determine blood glucose concentrations (12). Prediabetes status (yes/no) was determined based upon a fasting blood glucose measurement ≥100 mg/dL (15). The standard definition for diabetes based upon fasting blood glucose is a fasting blood glucose measurement ≥126 mg/dL (15). In the analyses, models predicting prediabetes thus define the outcome as individuals with prediabetes or diabetes.

### Assessment of predictors

Demographic information was collected during the APCAPS by a trained interviewer via questionnaire in the villages (12). Data on physical activity over the past week were collected by semiquantitative questionnaires previously adapted and evaluated for use in this population (16). The questionnaires also collected information on use of a ration card or a public distribution system card (yes/no), inability to walk (yes/no), alcoholic beverage consumption (g/d), and tobacco use (yes/no). Further, the data set contained information to denote individuals from the same family. This information would become important and was accounted for statistically when the generalized linear mixed model (GLMM) was explored as an analysis technique in the current study. The GLMM adjusts for both the potential correlation of observations due to the genetic effect of familial relations, and the potentially related dietary consumption within families.

### Statistical methods

#### ML primer

ML is beginning to find its way into nutrition studies (17, 18), and in practice can be broadly characterized by several cornerstone concepts (19). First, a modeling approach or algorithm is selected based on the type of data under study, scientific question at hand, and relevant data and modeling assumptions of importance. Second, the overall data set is typically split into “training” and “test” data sets according to a proportion chosen by the investigator (common ratios include 50:50, 80:20, and 85:15). The training data set is then used to fit, build, or “train” predictive models, whereas the test data set is held entirely aside, and only later used to test the models that were fit using the training data. The resulting performance of the models using the “fresh” test data set, often captured by AUC and other performance metrics, is then noted as a means of understanding how well the original predictive models describe the potential association between a set of predictors and an outcome of interest. This split between training and testing encapsulates a critical element of ML, wherein algorithms/models are deployed to discover existing relations between covariates and a specified outcome in the training data, and their performance on new data can be quantified through testing on new observations.

#### Data preparation

In order to assess the generalizability of each of the models explored in this study, we randomly sampled 85% of the families from the cohort to create a training data set to ensure a high volume of observations for fitting the initial models. This random split in noncorrelated data settings is typically done “by individual.” In this study, the split is performed “by family” so as to avoid any overlap across the training and test data sets of individuals who might be in the same family (and thus with potentially correlated outcomes). This is especially important in realistically assessing the performance of the GLMM, for reasons which will be further developed in the corresponding methods subsection. The test data, comprised of individuals from families entirely separate from individuals and families in the training data set, are held out in order to assess the performance of the predictive models that are built solely from individuals and families in the training data set. That is, we used the test data to assess how the models performed on individuals they had never screened before. Further, in a real-life prediabetes screening scenario, screening independent individuals instead of entire family units may be the most realistic occurrence. In order to simulate this scenario from the held-out test data under study, the test data in the analyses for this study contained 1 randomly selected individual from each of the remaining 15% of families.

#### Modeling overview

Six algorithms or models were explored in the prediction of the outcome prediabetes status (yes/no). Although overall diet quality was primarily accounted for using the GDQS, models were also fit using the daily totals for each GDQS food group (g/d). In addition to poor diet, other risk factors commonly associated with T2D include lack of exercise and smoking, among others, and so covariates including age, sex, hours sedentary, alcoholic beverage consumption, whether able to walk, use of ration cards, and tobacco use were considered in the models along with GDQS food groups or GDQS score. We developed the following models to predict the outcome prediabetes status (yes/no).

##### Random guessing model

This model was used to establish baseline performance. We first measured the prevalence of prediabetes in the training set and then randomly drew from a uniform (0, 1) distribution. Where *p* = prevalence of prediabetes in the training set, all sampled values in the range [0, *p*] were classified as having prediabetes or diabetes, whereas values in the range (*p*, 1] were classified as not having prediabetes or diabetes.

##### Logistic regression model

This model utilizes a parametric form to compute a predicted probability of prediabetes status for each participant. Models were fit to maximize the likelihood in the training set with regard to the prediabetes outcome (20). Logistic regression models were implemented using the *caret* package in R version 4.0.5 (R Core Team).

##### Least absolute shrinkage and selection operator regression

This model was used to correct for potential overfitting by the logistic regression model. Least absolute shrinkage and selection operator (LASSO) makes special use of shrinkage to enhance performance through an internal variable selection technique (21). The values of the model's parameter λ which minimize binomial deviance were determined through Five-fold cross validation on the training data set. The current implementation of LASSO used a logistic model for the binary prediabetes outcome. This model was implemented using the *glmnet* package in R version 4.0.5.

##### Elastic net

The aforementioned LASSO regression technique is a special case of the elastic net (EN) algorithm, where the parameter α is set equal to 1 (22). LASSO tends to select only 1 (or few) from a group of correlated predictors, whereas EN has greater facility to retain a larger number of correlated predictors, which can be a desirable model selection feature in data containing possibly related questionnaire items. As in the LASSO model, we implemented a logistic model for EN in the present work. Five-fold cross validation was utilized to assess the optimal value of λ using the *caret* package in R version 4.0.5.

##### Random forest

This binary classification model was developed using a nonparametric ML technique based upon an aggregation of many decision trees (23). We utilized 500 trees, a minimum node size of 1, and 25 features used per tree in our implementation of the random forest, chosen a priori. This algorithm was implemented using the *randomForest* package in R version 4.0.5.

##### Generalized linear mixed-effects model

The GLM, random forest, and regularization (LASSO, EN) methods aforementioned each assume independent and identically distributed outcome data (19). However, most commonly used ML methods do not account for correlated outcomes (whether longitudinal or clustered), with very few results to remedy this to date (24–26). The prediabetes data under study exhibit clear within-family correlation (calculation is given in the Results section), so the GLMM was explored as an option to account for nonindependent observations within family clusters. GLMMs include random intercepts for family-level effects and thus allow for estimation of and adjustment for correlation between individuals within the same family (27). Thus, in keeping with (frequentist) statistical theory on correlated outcomes, when using the GLMM, the data are first analyzed according to the original study design (clustered by family) in the training step. Only the fixed-effects estimates gained from this step (which are now appropriately adjusted for intrafamily correlation) are then used to build the prediction equation. That is, in order to avoid introducing bias in the training step by assuming responses within-families are not correlated when they truly are, we continued to use the GLMM to model such correlation structure via the random effects for family unit, in the training data set. However, we did not carry the random effects into the test step, and instead only carried forward the resulting adjusted fixed-effects estimates from the GLMM fitted to the training data, to form the prediction model (which ultimately took the form of a GLM). This prediction model was then assessed using the test data set.

Recall that the training/test data split was done by family to avoid overlap. This is particularly important when using the GLMM, because the estimation of random (family) effects in the training step should be based on data from individuals in family clusters that are entirely separate and independent from individuals in the test data, lest the predictive model enjoy a boost in performance simply based on familial correlation shared between observations in the training and test data sets. This is not a problem in contexts where it is expected that new observations will come from known clusters, but this is not a realistic scenario in our study. In summary, GLMMs were fit to the training data, and the resulting intracluster correlation (ICC)-adjusted fixed-effects estimates were then used as the prediction equation (essentially a GLM, after dropping the random effects for the new, predictive model). The performance of this predictive model was then assessed using the independent test data set. All GLMMs were fit using the *lme4* package in R version 4.0.5, using a penalized iteratively reweighted least-squares algorithm.

#### Model covariates and selection criteria

To establish baseline performance of the GDQS and age as predictors, 2 separate models were fit via the GLM using each of these features as the only predictor, respectively. A GLM including both age and GDQS together as the only 2 predictors was also fit. As a comparison, a GLMM was fit with both age and GDQS together, as well. It is important to note that the GLMM was not solely using covariate (age, GDQS, etc.) data, but was also using the prediabetes and diabetes status of other family members within a family cluster. A GLMM including age and the GDQS food group totals (g/d) as fixed effects was also fit. Finally, all models (GLM, LASSO, EN, random forest, GLMM) were assessed using the GDQS food group totals and the other previously described covariates as inputs. The LASSO, EN, and random forest models were only assessed using this full set of covariates because of their ability to perform feature reduction and the random forest's ability to handle nonlinear interactions between many covariates. Note that all models initially produce a predicted probability, rather than the binary outcome, as the output. The cutoff probability used to create a binary classification for the calculation of sensitivity and specificity was the prevalence of prediabetes in the training set (0.217).

For the GLM, LASSO, and random forest models, we also utilized the Synthetic Minority Oversampling Technique (SMOTE) resampling method to retrain select algorithms with a more balanced distribution of the outcome (28). We identified the model with the highest AUC on the test data set. We also reported sensitivity and specificity. The 95% CIs for the AUCs were computed using the DeLong method to compare related receiver operating characteristic curves (29).

## Results

### Participant characteristics

About 21% of the study population had prediabetes or diabetes (Table 1). The mean GDQS for all participants was 19.0 out of 42.5 points, and this value did not differ greatly between individuals who had prediabetes or diabetes and individuals who did not have prediabetes or diabetes. The distribution of the GDQS was approximately normal within both groups. The mean age of participants was 36 y with a range from 18 y to 85 y. The full data set contained 1728 unique families with 1–10 individuals in each family. The median family size was 3 individuals. The data set contained fewer individuals in the age range of ∼30–35 y than for all other ages. This may be an artifact of the sampling strategy used for the original study, because the age of the parents and siblings of the index children split into a bimodal distribution, peaking at ∼25 and 45 y of age.

**FIGURE 2:**
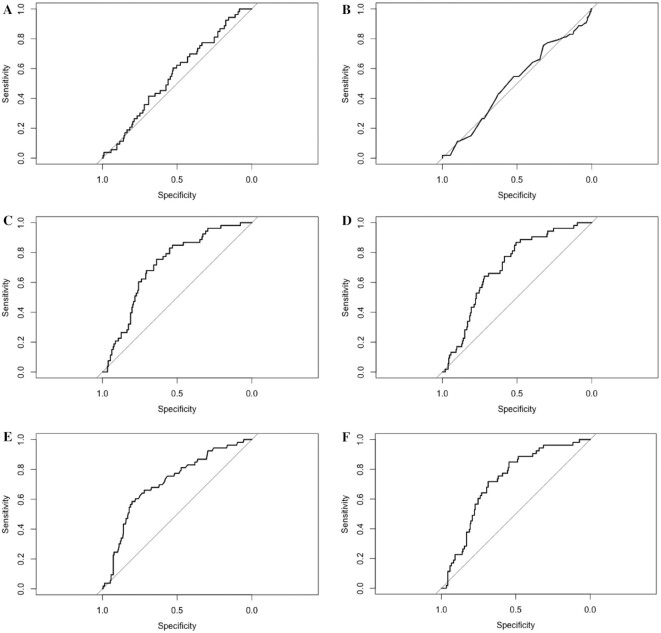
Receiver operating characteristic curves for the random guessing model (A), GLM with Global Diet Quality Score alone (B), GLM with all covariates (C), least absolute shrinkage and selection operator with all covariates (D), random forest with all covariates (E), and generalized linear mixed model with all covariates (F). GLM, generalized linear model.

**TABLE 1:** Selected characteristics of participants at wave 3 in the Andhra Pradesh Child and Parent Study^1^

Characteristics	No prediabetes^2^	Prediabetes or diabetes^3^	Overall
Participants	4440 (79)	1215 (21)	5655 (100)
Age, y	34.6 ± 13	41.1 ± 14	35.6 ± 14
Women	2099 (47.3)	534 (44.0)	2633 (46.6)
Ever use of tobacco^4^	1060 (23.9)	378 (31.1)	1438 (25.4)
Alcoholic beverage consumption, g/d	240 ± 701	344 ± 956	262 ± 764
Unable to walk^5^	254 (5.7)	150 (12.3)	404 (7.1)
Use of rations card	3117 (70.2)	687 (56.5)	3804 (67.3)
Time spent in sedentary activities, h/d	5.51 ± 3.4	5.79 ± 3.6	5.57 ± 3.4
Global Diet Quality Score	19.1 ± 3.6	18.9 ± 3.7	19.0 ± 3.6

^1^ *n* = 5655. Values are means ± SDs or *n* (%).

^2^Absence of prediabetes was defined as a fasting blood glucose concentration <100 mg/dL.

^3^Prediabetes and/or diabetes includes individuals with a fasting blood glucose concentration ≥100 mg/dL.

^4^Tobacco use is defined as having reported ever smoking, chewing, or snuffing tobacco products.

^5^Unable to walk responses exclude reasons related to shortness of breath.

The corresponding summary characteristics for both the training and test data are similar to those of the overall study population. Although individuals with prediabetes or diabetes in the testing set had a higher average GDQS (representing higher diet quality) than their counterparts without prediabetes or diabetes, this difference was not statistically significant (**Supplemental Table 2**). The prevalence of prediabetes or diabetes in the training and test data was ∼21%.

### Tuning of models

The implementation of EN determined that the best value of α was 1.0, which is equivalent to LASSO, as mentioned in the Methods. The optimized value of λ for the LASSO model was 0.01 when using GDQS food groups (g/d) among the other covariates mentioned as predictors. The EN implementation used a slightly different value of λ, based on minor differences between the functions used, so the performance reported was not identical. The LASSO model set many covariates to 0 but kept the food groups “red meat” and “refined grains and baked goods,” along with the covariates age, sex, and inability to walk. The number of trees used in the random forest was set a priori to the default 500 and was not tuned for this analysis.

### Predictive prediabetes model results

AUC values >0.70 for classifying the prediabetes outcome on the testing data were obtained from several models, including the fully adjusted GLMM with a random intercept for family (Table 2), which performed slightly better than most. The GLM including only GDQS as a predictor achieved an AUC lower than the random guessing model. On the other hand, a GLMM including age and GDQS as fixed effects achieved an AUC of 0.71 (95% CI: 0.64, 0.78), similar to the GLM using only age and GDQS as predictors with an AUC of 0.71 (95% CI: 0.64, 0.78). When we included additional predictors—sex, alcoholic beverage consumption, hours of sedentary activity, tobacco use, use of a rations card, and inability to walk—as fixed effects, the highest AUC of 0.72 (95% CI: 0.65, 0.79) was achieved by the GLMM. Several models achieved a lower AUC on the training set than on the test set (when we would typically expect the opposite); however, the CIs for train and test AUCs overlapped in these cases, except for the random forest model. The GLMM AUC on the training data set was higher than for the test set owing to this model's capacity to leverage prediabetes and diabetes status among individuals within the same family cluster in the training set, whereas the test set consisted only of individuals that were randomly sampled from the held-out test data [recall that no random effects (adjustment for family correlation) are performed in the test step for this model]. The implementation of SMOTE for the random forest, GLM, LASSO, and EN did not yield significant performance gains on our data, so these results are not presented. Figure 2 shows the receiver operating characteristic curves for the models on the test data.

**TABLE 2:** Performance metrics of select models for predicting prediabetes^1^

Algorithm	Predictors	Train AUC (95% CI)	AUC (95% CI)	Sensitivity	Specificity
Random guessing	NA	0.531 (0.512, 0.551)	0.556 (0.473, 0.639)	0.811	0.246
GLM	Age	0.635 (0.616, 0.653)	0.702 (0.629, 0.774)	0.774	0.570
GLM	GDQS	0.515 (0.495, 0.534)	0.511 (0.423, 0.598)	0.547	0.488
GLM	Age and GDQS	0.636 (0.617, 0.654)	0.709 (0.640, 0.779)	0.774	0.575
GLM	Age, GDQS food groups, hours sedentary, alcoholic beverage consumption, unable to walk, use of rations card, sex, tobacco use	0.654 (0.635, 0.672)	0.716 (0.645, 0.787)	0.755	0.600
GLMM	Age and GDQS, family random intercept	0.878 (0.867, 0.889)	0.710 (0.640, 0.779)	0.755	0.599
GLMM	Age and GDQS food groups, family random intercept	0.873 (0.861, 0.884)	0.711 (0.642, 0.781)	0.793	0.594
GLMM	Age, GDQS food groups, hours sedentary, alcoholic beverage consumption, unable to walk, use of rations card, sex, tobacco use, family random intercept	0.872 (0.861, 0.883)	0.722 (0.652, 0.792)	0.717	0.662
LASSO	Age, GDQS food groups, hours sedentary, alcoholic beverage consumption, unable to walk, use of rations card, sex, tobacco use	0.644 (0.625, 0.663)	0.705 (0.633, 0.776)	0.774	0.580
Elastic net (α = 1)	Age, GDQS food groups, hours sedentary, alcoholic beverage consumption, unable to walk, use of rations card, sex, tobacco use	0.641 (0.626, 0.659)	0.700 (0.627, 0.772)	0.774	0.570
Random forest	Age, GDQS food groups, hours sedentary, alcoholic beverage consumption, unable to walk, use of rations card, sex, tobacco use	1.000 (1.000, 1.000)	0.705 (0.633, 0.776)	0.774	0.517

1 AUC, area under the receiver operating characteristic curve; GDQS, Global Diet Quality Score; GLM, generalized linear model; GLMM, generalized linear mixed model; LASSO, least absolute shrinkage and selection operator; NA, not applicable.

## Discussion

### Model performance

In this cohort of 5655 participants from rural South India, we found that a GLM, LASSO, random forest, and GLMM with a random effect for family cluster all demonstrated adequate predictive capability for identifying prediabetes using only predictors derived from questionnaire data. The GLMM performed overall similarly to the other ML methods explored in this study while also incorporating potential within-family correlations between observations in the training data. Although the GDQS on its own did not perform strongly as a classifier, several models including the GDQS as well as models including the GDQS food groups achieved AUCs >0.70. The GLMM using age and GDQS as predictors obtained an AUC only 0.001 lower than the GLMM using age and GDQS food groups as predictors, suggesting that accounting for the GDQS in either form leads to similar performance in this task, although using the food group daily totals provides the model with more information about specific components of the diet. We found, not surprisingly, that age was a strong predictor of prediabetes within this cohort, with a GLM using age as the sole predictor achieving an AUC >0.70. Although the addition of other predictors improved model performance, an algorithm consisting of fixed effects for just age and GDQS as predictors performed well as a potential low-cost screening method for identifying prediabetes with AUCs >0.70 in the test set.

Although age was a stronger predictor than the GDQS for prediabetes, the GLM that included both age and GDQS slightly outperformed the GLM including age alone, although the difference in AUC was only ∼0.01. Still, these results reflect the importance of both age and diet quality as risk factors for developing T2D. Surprisingly, a GLM using GDQS alone resulted in a lower AUC than the random guessing model. This may be because the GDQS was developed primarily to identify nutrient adequacy and chronic disease risk among nonreproductive and nonlactating women of reproductive age (8). As such, cutoffs for assigning scores for GDQS food groups may need to be modified for other age groups and for men. This may also underscore the importance of adjusting for additional factors beyond diet quality alone if a researcher wishes to use diet in predicting diabetes risk. When examining the estimated coefficients of the individual food groups in the GLMM, it is worth noting that none of the individual food groups were significantly associated with prediabetes (**Supplemental Table 3**). Further, the dimension reduction resulting from the LASSO model kept only the *1*) red meat and *2*) refined grains and baked goods food groups. Still, given that the GDQS was developed as a simple tool based on food groups rather than nutrient composition tables, the ease of collecting this metric highlights its utility as an initial low-cost screening tool for identifying risk of chronic diseases including diabetes.

Given the global applicability of the GDQS, its utility is especially significant in LMICs and rural regions where resources are scarce. Screening large groups of individuals may often be difficult owing to lack of access to health care facilities, and even when such facilities are available, other logistical constraints such as proper refrigeration of blood samples and expensive diagnostic tests may prevent individuals from being diagnosed. Further research can assess whether consumption of certain component food groups of the GDQS is associated with diabetes risk in ways distinct from the overall score and how these associations may vary in different populations.

### Accounting for clustered data

The highest AUC in the test set was achieved by the GLMM with all covariates included. That is, our analyses showed that including family as a random intercept while fitting a model provides a statistically valid approach to accommodating family-cluster correlated data, while maintaining comparable performance in the development of a predictive model for classifying prediabetes in this population in rural South India. Although the performance of the GLMM was not significantly higher than that of other methods, there are other statistical advantages to properly adjusting for ICC when fitting a model on data with known clustering, in the ML setting. Because family is accounted for when fitting the model to these data, the fixed effects produced are thus ones which are adjusted for correlation within family. Of note, the ICC values were low at ∼0.2, which may be due to the large range of ages within the family (intergenerational members). The ICC is calculated by dividing the random effect variance by the total variance from the model fit. The within-cluster variance is assumed to be equal to π^2^/3 for the random intercept logistic model (30).

The clustering in our data also explains why the AUC of the GLMM in the training data is much higher than those of other models, aside from the random forest. In the fully adjusted model, the GLMM obtained an AUC of 0.87 (95% CI: 0.86, 0.88). This is because the GLMM, by modeling the correlation structure within each family via random effects and then adjusting for such in the estimation of fixed effects (predictors), was able to properly account for the clustering of families in a way that the other algorithms were not designed to do. In this way, the GLMM made use of an additional, important source of information in the estimation procedure. We expect that the GLMM would lead to even greater performance gains in the testing data if individuals in the testing data came from families in the training data, but this scenario may not be reasonable to implement in practice for the current problem. In studies such as the present one, it is more realistic to assume that new individuals being screened will come from new clusters (in our case, families) that are completely separate from the training set, rather than clusters with existing estimation of random effects from training. Future work can continue to explore methods which appropriately account for clustering in observations from new clusters.

### Strengths, limitations, and future work

The strengths of the current analysis include the availability of India-specific dietary data, laboratory measures, family structure of participants, and the large sample size. However, some important limitations need to be considered. First, our study was cross-sectional in nature and therefore we cannot establish a causal effect of diet quality on disease. Still, our ability to develop an algorithm to classify prediabetes using cross-sectional data is noteworthy. In addition, the use of an FFQ to measure diet can introduce some degree of measurement error. However, the FFQ was validated against 24-h recalls and accounted for seasonal foods. Similarly, using fasting blood glucose to define the outcome may introduce measurement error, because there is no way to confirm that all measurements were taken after fasting, and this measurement may not provide the full context in diagnosis that additional information about an oral-glucose-tolerance test or glycated hemoglobin would provide.

A further limitation of this work is the geographic distribution of the study population. Because all APCAPS participants reside in the same region of South India, rural Hyderabad, further work is necessary to validate these findings in other parts of India. Future research should compare whether adjusting for the geographic region leads to performance gains in a model deployed on a national or international scale, because prevalence of the outcome is likely to differ by region. The utility of the GDQS in the present modeling efforts provides support for the feasibility of this additional work because the GDQS metric can provide a single, standardized measure of diet quality even for individuals in different regions of the country. The use of GDQS food groups also provides the model with more information about the components of an individual's diet while requiring fewer questions to measure than a complete FFQ. Finally, the GDQS was developed for use in nonpregnant, nonlactating women of reproductive age and further validation is needed for other age groups, pregnant/lactating women, and men. Nevertheless, this global metric of diet quality showed promising results when combined with age, family, and other covariates to classify prediabetes using a variety of algorithms. Future work needs to explore whether altering the point values assigned to consumption totals in each GDQS food group for other age groups would improve model performance for this outcome. In addition, future work can explore the impact of outlier values in certain food groups on prediction accuracy, because they were kept in the overall data set.

Additional limitations of the study relate to the availability of data and computational costs of further analyses. Although we were able to define models with adequate classification ability in the test data (AUC >0.70), additional work is required to confirm the stability of these findings. This concern is raised especially when considering the low AUC values of some models on the training data. It is possible that the testing data were significantly different from the training data in some way owing to randomness in the splitting process, leading to lower performance in the training data. Splitting by family can lead to inconsistencies between the training and test sets if there are large differences between the families selected for each set. This could then be reflected in the performance of models estimated then tested on these sets, respectively. By contrast, the random forest model achieved perfect classification in the training data but achieved an AUC of 0.71 in the test data. This strongly suggests the presence of overfitting during training of the random forest. A simulation study assessing the performance of the same algorithms across many random splits of the training and test data can help to understand the stability of these classifiers. As an additional consideration, the testing performed in the current study involved individuals from the same study as those used to train the model came from (although separate individuals and families were used in the training/testing data), so it is possible that the classifier learned to detect patterns specific to our cohort rather than generally applicable trends. External validation must be performed in future work to gain a more concrete understanding of the generalizability of these results.

Future classifiers may also wish to make use of other factors associated with diabetes risk, such as BMI, waist-to-hip ratio, or other physical measurements. Whereas our study focused specifically on factors that could be measured using only a questionnaire, it may be necessary to record some physical measurements to obtain more accurate assessments of diabetes risk. Our analyses demonstrated that age was a very strong predictor of this outcome and was necessary to improve the performance of the GDQS for this outcome, and it is feasible that additional measurements, rather than any specific algorithm, will most considerably improve the performance of future classifiers.

### Conclusion

Results from our study confirm that several models including age and a global measure of diet quality can classify prediabetes with reasonable discrimination. The GLMM, which models the dependence between clustered observations and utilizes the resulting information in its estimates of fixed effects, could prove an additional, helpful tool within the array of ML methods when encountering correlated data. A facility for avoiding possible misspecification of models when encountering cluster-correlated data in similar predictive ML tasks may be a worthy contribution the GLMM offers, within the context of diet quality and disease studies. The random forest model provides the flexibility of a nonparametric approach for modeling disease risk. The LASSO provides the benefit of identifying the features most important in predicting the outcome and can help reduce the number of predictors needed for the screening.

Given the high burden of diabetes, the use of low-cost and simple-to-implement screening tools as a first step in identifying high-risk groups shows considerable promise. Future studies need to examine the utility of the GDQS in screening for other noncommunicable diseases.

## Supplementary Material

nxab281_Supplementary_Tables_1_3

## Notes

Funding for the research was provided by FHI Solutions, recipient of a Bill & Melinda Gates Foundation grant to support Intake – Center for Dietary Assessment. Andhra Pradesh Children and Parent Study data collection was funded by Wellcome Trust grants 083707, 084774, and 084754.

Author disclosures: TTF is an Associate Editor for the Journal of Nutrition and played no role in the Journal’s evaluation of the manuscript. All other authors report no conflicts of interest.

Supplemental Tables 1–3 are available from the “Supplementary data” link in the online posting of the article and from the same link in the online table of contents at https://academic.oup.com/jn.

SNB and EL contributed equally to this work as senior authors.

Published in a supplement to *The Journal of Nutrition*. Publication costs for this supplement were funded by the Bill & Melinda Gates Foundation in conjunction with FHI Solutions, recipient of a Bill & Melinda Gates Foundation grant to support Intake—Center for Dietary Assessment. The opinions expressed in this publication are those of the authors and are not attributable to the sponsors or the publisher, Editor, or Editorial Board of *The Journal of Nutrition*. The Supplement Coordinators for the supplement publication were Megan Deitchler, Intake—Center for Dietary Assessment at FHI Solutions, Washington, DC; and Sabri Bromage, Harvard TH Chan School of Public Health, Boston, MA.

The GDQS research initiative was launched by Intake – Center for Dietary Assessment. The research was led by Harvard T.H. Chan School of Public Health, Department of Nutrition and carried out in collaboration with researchers at the National Public Health Institute (INSP), Mexico. Funding for the research was provided by FHI Solutions, recipient of a Bill & Melinda Gates Foundation grant to support Intake – Center for Dietary Assessment.

Abbreviations used: APCAPS, Andhra Pradesh Children and Parent Study; AUC, area under the receiver operating characteristic curve; EN, elastic net; GDQS, Global Diet Quality Score; GLM, generalized linear model; GLMM, generalized linear mixed model; ICC, intracluster correlation; LASSO, least absolute shrinkage and selection operator; LMIC, low- and middle-income country; ML, machine learning; SMOTE, Synthetic Minority Oversampling Technique; T2D, type 2 diabetes.
