# Supplementary material for: Exploration of Machine Learning and Statistical Techniques in Development of a Low-Cost Screening Method Featuring the Global Diet Quality Score for Detecting Prediabetes in Rural India
Source: J Nutr. 2021 Oct 23;151(Suppl 2):110S–118S. doi: 10.1093/jn/nxab281 (PMC8542097; doi:10.1093/jn/nxab281)
Supplement: nxab281_Supplementary_Tables_1_3 [file nxab281_supplementary_tables_1_3.docx]

**Exploration of Machine Leaning and Statistical Techniques in Development of a Low-Cost Screening Method Featuring the Global Diet Quality Score for Detecting Prediabetes in Rural India**

Nick Birk

Online Supplementary Material

**Supplementary Table 1.** **Scoring system for Global Diet Quality Score food groups*^1^***

| **Food Group** | **Score Cutoffs (g/d)** | | | | **Points** | | | |
| --- | --- | --- | --- | --- | --- | --- | --- | --- |
|  | **Low** | **Medium** | **High** | **Very high*^2^*** | **Low** | **Medium** | **High** | **Very high*^2^*** |
| Legumes | <10 | 10-39 | >39 | NA | 0 | 2 | 4 | NA |
| Nuts and seeds | <4 | 4-16 | >16 | NA | 0 | 2 | 4 | NA |
| Whole grains | <4 | 4-16 | >16 | NA | 0 | 1 | 2 | NA |
| Liquid oils | <2 | 2-7.5 | >7.5 | NA | 0 | 1 | 2 | NA |
| Dark green leafy vegetables | <10 | 10-39 | >39 | NA | 0 | 2 | 4 | NA |
| Fish | <16 | 16-63 | >63 | NA | 0 | 1 | 2 | NA |
| Deep orange fruits | <28 | 28-114 | >114 | NA | 0 | 1 | 2 | NA |
| Citrus fruits | <18 | 18-74 | >74 | NA | 0 | 1 | 2 | NA |
| Other fruits | <26 | 26-106 | >106 | NA | 0 | 1 | 2 | NA |
| Low-fat dairy | <35 | 35-139 | >139 | NA | 0 | 1 | 2 | NA |
| Poultry and game meat | <12 | 12-48 | >48 | NA | 0 | 1 | 2 | NA |
| Eggs | <7 | 7-28 | >28 | NA | 0 | 1 | 2 | NA |
| Cruciferous vegetables | <11 | 11-44 | >44 | NA | 0 | 0.25 | 0.5 | NA |
| Deep orange vegetables | <10 | 10-39 | >39 | NA | 0 | 0.25 | 0.5 | NA |
| Deep orange tubers | <14 | 14-57 | >57 | NA | 0 | 0.25 | 0.5 | NA |
| Other vegetables | <26 | 26-106 | >106 | NA | 0 | 0.25 | 0.5 | NA |
| High-fat dairy | <35 | 35-139 | 140-734 | >734 | 0 | 1 | 2 | 0 |
| Red meat | <12 | 12-48 | >48 | NA | 0 | 1 | 0 | NA |
| Juice | <35 | 35-141 | >141 | NA | 2 | 1 | 0 | NA |
| Processed meat | <8 | 8-31 | >31 | NA | 2 | 1 | 0 | NA |
| White roots and tubers | <25 | 25-100 | >100 | NA | 2 | 1 | 0 | NA |
| Refined grains and baked goods | <7 | 7-28 | >28 | NA | 2 | 1 | 0 | NA |
| Sugar-sweetened beverages | <52 | 52-207 | >207 | NA | 2 | 1 | 0 | NA |
| Sweets and ice cream | <11 | 11-45 | >45 | NA | 2 | 1 | 0 | NA |
| Purchased deep fried foods | <10 | 10-40 | >40 | NA | 2 | 1 | 0 | NA |

*^1^* NA, Not applicable

*^2^* The “very high” category only applies to the high-fat dairy food group

**Supplementary Table 2. Selected characteristics of participants in the training dataset (n=4837) and the testing dataset (n=260) at wave three in the Andhra Pradesh Child and Parent Study*^1^***

|  | **Training dataset** | | | **Testing dataset** | | |
| --- | --- | --- | --- | --- | --- | --- |
|  | No prediabetes*^2^* | Prediabetes or Diabetes*^3^* | Overall | No prediabetes*^2^* | Prediabetes or Diabetes*^3^* | Overall |
| Participants, *n* (%) | 3786 (78.3) | 1051 (21.7) | 4837 | 207 (79.6) | 53 (20.4) | 260 |
| Age, years | 34.6 ± 14 | 41.0 ± 14 | 36.0 ± 14 | 35.0 ± 13 | 44.4 ± 11 | 36.9 ± 13 |
| Women, *n* (%) | 1785 (47.1) | 462 (44.0) | 2247 (46.5) | 104 (50.2) | 26 (49.0) | 130 (50.0) |
| Ever use of tobacco*^4^*, *n* (%) | 882 (23.3) | 334 (31.8) | 1216 (25.1) | 54 (26.1) | 11 (20.8) | 65 (25.0) |
| Alcoholic beverage consumption, g/d | 242 ± 721 | 323 ± 764 | 260 ± 732 | 182 ± 480 | 386 ± 844 | 224 ± 577 |
| Unable to walk*^5^*, *n* (%) | 215 (5.7) | 130 (12.4) | 345 (7.1) | 15 (7.2) | 5 (9.4) | 20 (7.7) |
| Use of rations card, *n* (%) | 2640 (69.7) | 593 (56.4) | 3233 (66.8) | 142 (68.6) | 29 (54.7) | 171 (65.8) |
| Time spent in sedentary activities, hr/d | 5.55 ± 3.4 | 5.76 ± 3.5 | 5.60 ± 3.4 | 5.13 ± 3.0 | 5.63 ± 3.1 | 5.23 ± 3.0 |
| Global Diet Quality Score | 19.1 ± 3.7 | 18.9 ± 3.7 | 19.0 ± 3.7 | 18.9 ± 3.5 | 19.0 ± 3.7 | 18.9 ± 3.5 |

*^1^* Values are expressed as means ± SD for all variables, except women, ever use of tobacco, unable to walk, and use of rations card which are expressed as total number and percentages.

*^2^* Absence of prediabetes was defined as a fasting blood glucose <100 mg/dL

*^3^* Prediabetes and/or diabetes includes individuals with a fasting blood glucose ≥100 mg/dL

*^4^* Tobacco use is defined as having reported ever smoking, chewing, or snuffing tobacco products.

*^5^* Unable to walk responses exclude reasons related to shortness of breath.

**Supplementary Table 3. Fixed effect exponentiated age-adjusted coefficients (95% confidence intervals) of the 25 food groups in the Global Diet Quality Score with prediabetes in the Andhra Pradesh Child and Parent Study*^1^***

| **Food component** | **Prediabetes (95% CI)** |
| --- | --- |
| Legumes | 1.00 (1.00, 1.01) |
| Nuts and seeds | 1.00 (0.996, 1.01) |
| Whole grains | 1.00 (0.997, 1.00) |
| Liquid oils | 1.00 (0.989, 1.01) |
| Dark green leafy vegetables | 0.997 (0.987, 1.01) |
| Fish | 1.00 (0.994, 1.01) |
| Deep orange fruits | 0.999 (0.973, 1.03) |
| Citrus fruits | 0.997 (0.990, 1.00) |
| Other fruits | 1.00 (0.999, 1.00) |
| Low-fat dairy*^2^* | NA |
| Poultry and game meat | 1.00 (0.996, 1.00) |
| Eggs | 1.00 (0.997, 1.01) |
| Cruciferous vegetables | 1.01 (0.988, 1.03) |
| Deep orange vegetables | 0.999 (0.973, 1.03) |
| Deep orange tubers | NA |
| Other vegetables | 1.00 (0.997, 1.00) |
| High-fat dairy | 1.00 (1.00, 1.00) |
| Red meat | 1.01 (0.999, 1.02) |
| Juice*^3^* | NA |
| Processed meat*^4^* | NA |
| White roots and tubers | 0.999 (0.989, 1.01) |
| Refined grains and baked goods | 1.00 (1.00, 1.00) |
| Sugar-sweetened beverages | 1.00 (0.999, 1.00) |
| Sweets and ice cream | 0.999 (0.995, 1.00) |
| Purchased deep fried foods | 0.997 (0.993, 1.00) |

*^1^* Values presented are age-adjusted exponentiated coefficient estimates and 95% Wald confidence intervals with Global Diet Quality Score food categories (and age) as fixed effects and family as a random intercept. CI, Confidence interval; NA, Not applicable

*^2^* Low-fat dairy was not consumed as all dairy consumed had at least 3% milk fat.

*^3^* Juice was combined with sugar-sweetened beverages because of the high amount of added sugar.

*^4^* Processed meat was not consumed in this cohort.
